# Clinical Impact of EUS-Guided Fine Needle Biopsy Using a Novel Franseen Needle for Histological Assessment of Pancreatic Diseases

**DOI:** 10.1155/2019/8581743

**Published:** 2019-02-03

**Authors:** Takuya Ishikawa, Hiroki Kawashima, Eizaburo Ohno, Hiroyuki Tanaka, Daisuke Sakai, Tadashi Iida, Ryo Nishio, Takeshi Yamamura, Kazuhiro Furukawa, Masanao Nakamura, Ryoji Miyahara, Senju Hashimoto, Masatoshi Ishigami, Yoshiki Hirooka

**Affiliations:** ^1^Department of Gastroenterology, Nagoya University Graduate School of Medicine, 65 Tsuruma-cho, Showa-ku, Nagoya, Aichi 466-8550, Japan; ^2^Department of Endoscopy, Nagoya University Hospital, 65 Tsuruma-cho, Showa-ku, Nagoya, Aichi 466-8550, Japan; ^3^Department of Liver, Biliary Tract and Pancreas Diseases, Fujita Health University Hospital, 1-98 Dengakugakubo, Kutsukake-cho, Toyoake, Aichi 470-1192, Japan

## Abstract

**Background and Aims:**

Several studies have shown the benefits of endoscopic ultrasound-guided fine needle biopsy (EUS-FNB) using a Franseen needle for histological assessment. However, studies focusing on pancreatic diseases are limited and the safety of this method has not been well assessed. We aimed to assess the current status and issues of EUS-FNB in the diagnosis of pancreatic diseases.

**Materials and Methods:**

We retrospectively reviewed 87 consecutive EUS-FNB specimens using either a 22-gauge Franseen needle (Group A, N = 51) or a conventional 22-gauge fine-needle aspiration needle (Group B, N = 36) for pancreatic diseases, and the diagnostic accuracy and safety were compared. Final diagnoses were obtained based on surgical pathology or a minimum six-month clinical follow-up.

**Results:**

Although the diagnostic accuracy for malignancy was 96.1% in Group A versus 88.9% in Group B, with no statistically significant difference (*P* = 0.19), the median sample area was significantly larger in Group A (4.07 versus 1.31mm^2^,* P* < 0.0001). There were no differences between the two needles in the locations from which the specimens were obtained. Adverse events occurred in one case (2%) in Group A (mild pancreatitis) and none in Group B with no statistical significance (*P* = 0.586). Although there was no case of bleeding defined as adverse events, 2 cases in Group A showed active bleeding during the procedure with increase in the echo-free space, which required CT scanning to rule out extravasation. Eventually, the bleeding stopped spontaneously.

**Conclusions:**

Given its guaranteed ability to obtain core specimens and comparable safety, and although the risk of bleeding should be kept in mind, EUS-FNB using a Franseen needle is likely to become a standard procedure for obtaining pancreatic tissue in the near future.

## 1. Introduction

In 1998, we first reported the potential for histological diagnosis with endoscopic ultrasound- (EUS-) guided tissue sampling [1]. Since then, EUS-guided tissue acquisition techniques have evolved. Recently, several new “core” needles for EUS-guided fine needle biopsy (FNB), in contrast to fine needle aspiration (FNA), have been developed for obtaining samples for histology. We have reported the initial experience with one such needle, a fork-tipped needle, in Canada [2]; the histological cores obtained with this needle yielded a definite diagnosis, even in cases with equivocal cytomorphology. In Japan, a needle with three novel symmetrical heels called a Franseen needle has become available for EUS-FNB. Several studies have already shown the benefits of EUS-FNB using the Franseen needle for histological assessment [3–5]; however, studies focusing on pancreatic diseases are limited. Furthermore, the safety of this method has not been well assessed, and concerns of an increased risk of bleeding or pancreatitis due to the unique shape of the needle remain. Therefore, we assessed the usefulness and safety of this novel Franseen needle compared with a conventional FNA needle and aimed to figure out the current status and issues of EUS-FNB for the histological diagnosis of pancreatic diseases.

## 2. Materials and Methods

### 2.1. Study Design

This was a retrospective study performed at a single tertiary care referral center (Nagoya University Hospital). Written informed consent was obtained from each patient or family (if the patient was deceased when obtaining the consent), and the study was performed with the approval of the ethics committee of Nagoya University Graduate School of Medicine.

### 2.2. Patients

We retrospectively reviewed 87 consecutive EUS-FNB specimens obtained from 82 patients using either an Acquire™ 22-gauge needle (Boston Scientific Co., Natick, MA, USA) (Group A, N = 51 specimens from 50 patients) or a conventional 22-gauge FNA needle (EZ shot 3 Plus™, Olympus Co., Ltd., Tokyo, Japan) (Group B, N = 36 specimens from 36 patients) to diagnose pancreatic diseases between October 2016 (when the Acquire needle became available in our facility) and March 2018. In this study, EUS-FNB was defined as the use of an EUS-guided tissue acquisition technique to obtain a sample for histology, regardless of the type of needle used. In our facility, EUS-FNB (or FNA) is not performed for pancreatic cancers that are surgically resectable based on radiological findings. Thus, the majority of the pancreatic lesions included in this study were unresectable pancreatic cancers, those that required differentiation between malignant and benign status, or those with atypical appearance on radiology. During the study period, there were 21 patients with possible autoimmune pancreatitis (AIP) who underwent EUS-FNB. However, as we have been conducting a prospective study (UMIN-CTR: UMIN000026692) evaluating the diagnostic ability of EUS-FNB and the Franseen needle was used for all suspected AIP cases, they were excluded from this study.

### 2.3. EUS-FNB Procedure

The EUS procedure was performed by one of three experienced endosonographers (> 250 EUS cases per year) using a linear-array endoscope (EG 580UT, Fujifilm Co., Ltd., Tokyo, Japan, or GF-UCT260, Olympus Co., Ltd., Tokyo, Japan).

**Figure 1:**
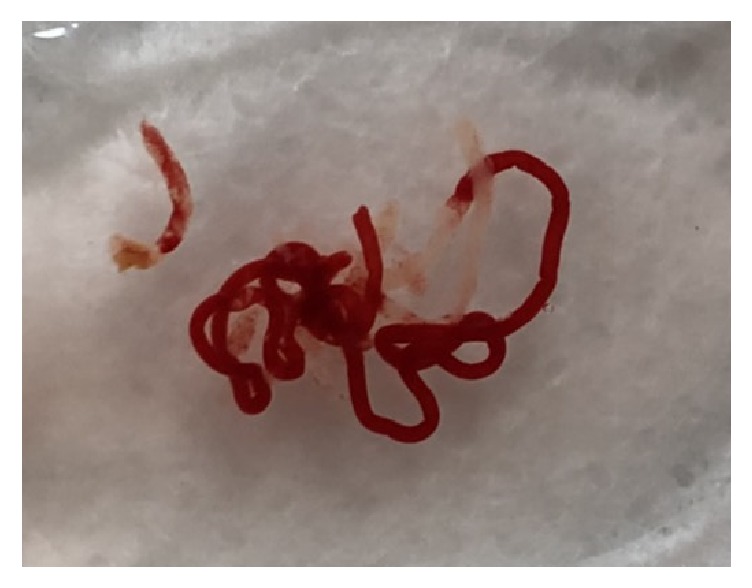
Fresh specimen obtained with a 22-gauge Franseen needle. Fragments of whitish tissue can be detected macroscopically between the blood clots.

While the patient was under conscious sedation, the EUS scope was inserted orally. The lesion was carefully observed in B-mode first and then in color Doppler mode before puncture to confirm that no major vessels were in the needle pathway. The selection of the needle was left to the discretion of the endoscopist, but, regarding the gauge of the needle, a 22-gauge needle was used as a standard method. A 25-gauge needle was used if the approach by the 22-gauge needle was difficult because of any reasons such as the location, size of the lesion, or surrounding vessels. A 19-gauge needle was used only when it was undiagnostic with a 22-gauge needle and the size and location of the lesion was technically suitable for the use of 19-gauge needle. When the Franseen needle was used, after the needle was inserted into the lesion, the stylet was slowly withdrawn (dry slow-pull technique) as the sample was obtained for all of the needle passes. When the conventional FNA needle was used, after puncture with the needle and stylet, the stylet was removed from the needle, and the specimens were collected under 20 mL negative pressure with 10 to 20 back-and-forth movements. For the procedure using a 22-gauge conventional FNA needle, only EZ shot 3 Plus was used during this study period. The collected specimens were removed for histological processing by slowly reinserting the stylet to express the entire sample directly into formalin. Because rapid on-site specimen evaluation (ROSE) is not available at our institution, the number of passes was left to the discretion of the endoscopist. In principle, the procedure was repeated until visible fragments of white/tan tissue were observed when the specimens were expressed into formalin (Figure 1), with a maximum of five passes.

### 2.4. Outcomes

The primary outcome in this study was the diagnostic ability for pancreatic cancer (sensitivity, specificity, and accuracy) with each needle (based on the results from total session of EUS-FNB), and the secondary outcomes were the total area of the specimen obtained with each needle and adverse events (AEs).

### 2.5. Histology Evaluation

All the collected specimens were sent for histological examination. After formalin fixation, the specimens were embedded in paraffin, sectioned, and subjected to hematoxylin-eosin (H&E) staining and appropriate immunostaining according to the suspected diagnosis. All the histological diagnoses were performed by two pathologists who were specialized in pancreatobiliary field at Nagoya University Hospital.

Final diagnoses were based on surgical pathology or a minimum six-month clinical follow-up. As mentioned above, EUS-FNB was not performed for radiologically resectable pancreatic cancers, and most of the cases with pancreatic cancer were unresectable in this study. Finally, 14 of 82 cases (including 11 pancreatic cancer cases which required differentiation from mass forming pancreatitis or showed atypical appearance on radiology) underwent surgery and the rest of the 68 cases were diagnosed based on clinical follow-up.

**Figure 2:**
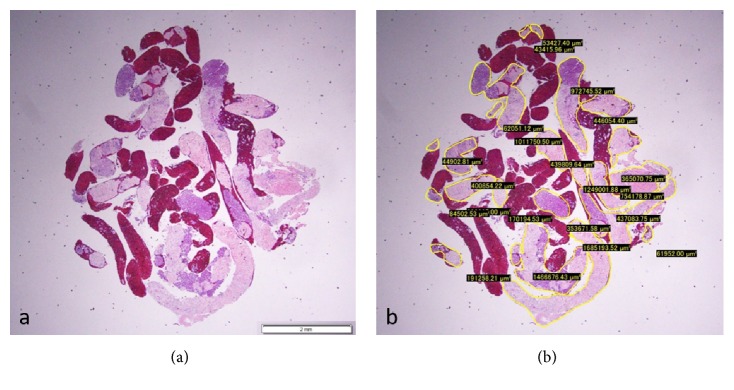
(a) Hematoxylin and eosin staining of a gross specimen obtained from the pancreas using a 22-gauge Franseen needle, viewed in a low-power field. (b) Measuring the area of the specimen, excluding the blood clots, using imaging software (CellSense).

The total area of the specimen obtained with each needle was also measured and compared under a photomicroscope using imaging software (CellSense, Olympus Co., Ltd., Tokyo, Japan) based on our previous report (Figure 2) [6].

### 2.6. Safety Evaluation

Any AEs were recorded and compared according to the lexicon for endoscopic AEs advocated by American Society of Gastrointestinal Endoscopy [7]. In addition, any incidents including bleeding that stopped spontaneously or with endoscopic therapy during the procedure were also recorded in this study.

### 2.7. Statistical Analysis

Statistical analyses were performed using SPSS Statistics 25.0 (SPSS, Inc., Chicago, IL, USA). The *χ*2 test and Fisher's exact test were used to compare categorical parameters, and the Mann-Whitney U test was used to compare continuous variables. Continuous parameters are presented as the median (IQR). A *P* value of less than 0.05 was considered statistically significant.

## 3. Results

### 3.1. Patient Characteristics

There were no differences between the two groups in the mean age or gender of the patients, the mean size of the lesions, and the final diagnoses (Table 1).

### 3.2. Histological Assessment

#### 3.2.1. Comparison of the Specimens Obtained by Two Needles

All but one of the 87 specimens in both groups were adequate for histological examination, with no difference in the mean number of passes (2 versus 2) (Table 2). There were no differences between the two needles in the locations from which the specimens were obtained; 27/51 (52.9%) samples were obtained from the pancreatic head or uncinate process with a Franseen needle with satisfactory results. The sensitivity, specificity, and accuracy for malignancy were 95.3%, 100%, and 96.1% in Group A versus 88.2%, 100%, and 88.9% in Group B, with no statistically significant difference (samples with atypical cells and those that were suspicious for malignancy were considered malignant). However, the median sample area of Group A was significantly larger than that of Group B (4.07 versus 1.31mm^2^,* P* < 0.0001). Although the selection of needles was left to the discretion of the endoscopist, when comparing the types of needles used in the first and second 9 months, the Franseen needle was used significantly more frequently in the second 9-month period (*P* = 0.001, Table 3).

**Figure 3:**
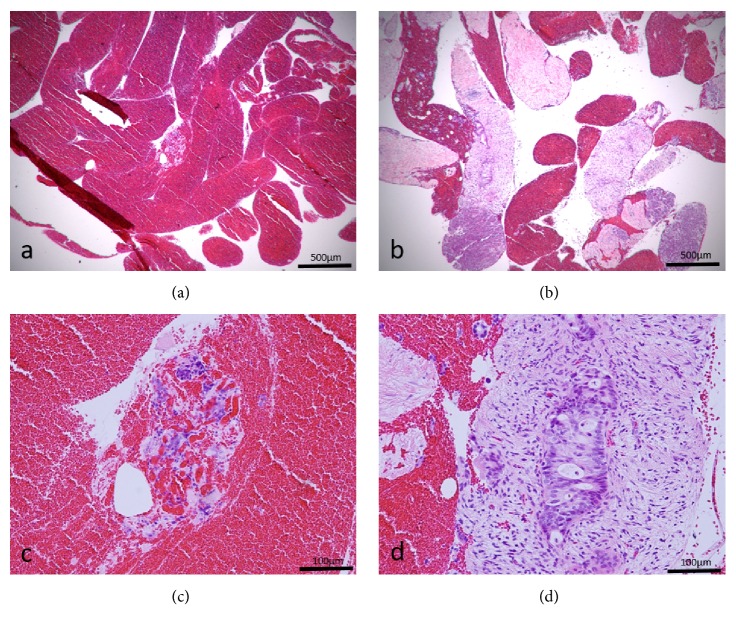
Specimens obtained from the same pancreatic cancer lesion using both a conventional fine needle aspiration (FNA) needle (a, c) and a Franseen needle (b, d). The specimen obtained by the FNA needle shows many blood clots (a), whereas the specimen obtained by the Franseen needle shows good core tissues with low-power field magnification. (c) Scattered atypical cells can be identified in the blood clots with high-power field magnification, but they are insufficient for a cancer diagnosis. (d) A component of atypical cells with enlarged nuclei in the fibrous stroma is detected with high-power field magnification, consistent with ductal carcinoma of the pancreas.

Of the 7 cases that were undiagnosed with the first session of EUS-FNB, 3 cases underwent repeat EUS-FNB (one in Group A using a 19-gauge Franseen needle and 2 in Group B using a 22-gauge Franseen needle), and all of them were finally diagnosed with adenocarcinoma (one case had a total of 3 sessions using the Franseen needle twice). The remaining 4 cases were diagnosed clinically without repeat EUS-FNB. Two cases which showed atypical cells in Group B also underwent repeat EUS-FNB using a 22-gauge Franseen needle. One case achieved definite diagnosis of adenocarcinoma (Figure 3), but the other one showed atypical cells again with the Franseen needle and was finally diagnosed as pancreatic cancer in combination with the clinical follow-up.

### 3.3. Safety Assessment

**Figure 4:**
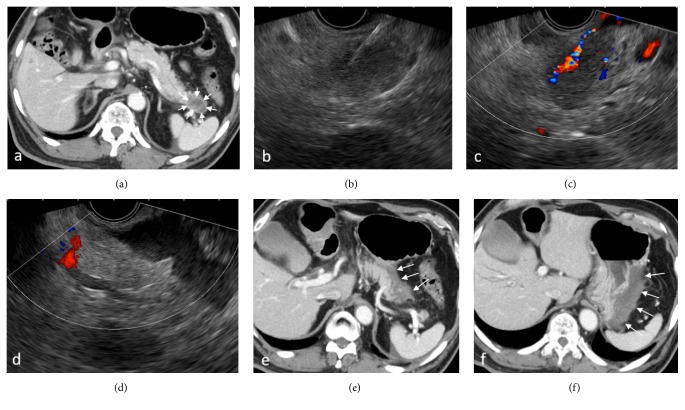
A patient who developed bleeding with endoscopic ultrasound-guided fine needle biopsy (EUS-FNB) using a Franseen needle. (a) Contrast-enhanced Computed Tomography (CT) scan showing a 3-cm hypovascular mass in the pancreatic tail (arrow). (b) Insertion of the needle under EUS guidance. (c) Active bleeding from the needle tract right was noticed under color Doppler mode after the withdrawal of the needle. (d) Increased echo-free space between the pancreas and stomach was identified. ((e), (f)) Contrast-enhanced CT scan was performed immediately after EUS-FNB. Hyperdense fluid collection suggesting hematoma was observed between the pancreatic tail and the greater curvature of the stomach (arrow).

Adverse events occurred in 1 of 51 sessions (2%) in Group A and none in Group B, with no significant difference (*P* = 0.685) (Table 4); mild pancreatitis occurred in a case of intraductal tubulopapillary neoplasm (ITPN) in Group A as a result of the intentional insertion of the needle into the pancreatic duct, but the patient recovered in a few days with conservative treatment. There was no case of bleeding defined as AE; however, bleeding from the puncture site occurred during the procedure with both types of needles (3 cases in Group A and 2 cases in Group B); although it stopped without intervention in all cases, 2 cases in Group A showed active bleeding under color Doppler after the withdrawal of the needle and an increase in the echo-free space between the pancreas and stomach, which required CT scanning to rule out extravasation (Figure 4 and supplementary video 1). The remaining one case in Group A and 2 cases in Group B showed pulsating bleeding from the puncture site on endoscopy, but, in all cases, the bleeding stopped spontaneously when the scope was changed from EUS to a front-viewing scope.

## 4. Discussion

EUS-guided FNA/biopsy is an established and widely used tissue sampling technique for the pancreas[8], and there is no doubt that this technique will provide promising results in the diagnosis of pancreatic diseases. However, the amount of specimens that can be obtained with a conventional FNA needle is limited and can preclude diagnoses that require detailed histological assessment, such as AIP [9] or neoplasms with atypical histology [10]. In this study, we have shown that the Franseen needle obtained significantly larger samples than were obtained with a conventional FNA needle, even from the pancreatic head or uncinate process, which have been difficult to sample with existing core needles [11–15]. It is noteworthy that although needle selection was left to the discretion of the endoscopist, the Franseen needle was used significantly more frequently than the conventional FNA needle in the second half of the study period, regardless of the suspected disease. It is reassuring to be able to confirm the presence of whitish tissue macroscopically with the naked eye when the sample is expressed from the needle. This will surely reduce the stress of the endosonographer and the pathologist when diagnosing the specimen. Recently, a prospective assessment of the performance of EUS-FNB using a Franseen needle for solid lesions including 51 specimens from various lesions was reported [16]. This study demonstrated a histologically superior sample with fewer passes compared to conventional FNA needles of the same size, and they concluded that an FNB exclusive approach to sampling all solid lesions appears feasible, supporting the results of the present study. In our study, there was no difference in the number of passes between a Franseen needle and a conventional FNA needle. However, the mean number of passes in both needles was only 2 and, considering the situation without ROSE in our facility, this number would be acceptable.

Regarding safety, the only AE was mild pancreatitis in Group A and there was no statistically significant difference in the number of AEs between the two groups. There was no case of bleeding defined as AE in both groups, but some cases showed bleeding during the procedure, and the risk of bleeding appeared to be slightly higher in the Franseen needle group. Although all cases of bleeding were treated without interventions such as IVR or blood transfusion, 2 cases in the Franseen needle group showed active bleeding on EUS and needed to undergo a CT scan to rule out intra-abdominal bleeding. El Hajj et al. [16] have reported that 2 out of 51 cases who underwent EUS-FNB using a Franseen needle developed a hematoma outside the gastric and duodenal walls. The hematomas were monitored for 10 minutes with stable appearance on EUS and did not result in clinically apparent adverse outcomes. Bang JY et al. [5] have also reported that one out of 30 patients who underwent EUS-FNB using a Franseen needle developed mucosal bleeding (arterial) which was treated by placement of 2 endoscopic clips. All of these reported bleeding events were mild and controllable; however, further assessment may be required in a larger cohort. At any hand, the risk of bleeding should be kept in mind when performing EUS-FNB, and it is important to confirm the absence of vessels in the needle pathway under color Doppler mode before puncture and observe the image carefully after withdrawal of the needle.

There are several limitations to this study. First, it was a retrospective study. Further prospective studies with a larger number of patients are necessary to confirm these results. Second, ROSE was not performed in this study. The sensitivity of EUS-FNA is known to depend on the availability of an on-site cytopathology assessment, which has been clearly demonstrated to significantly influence both the diagnostic accuracy and the proportions of indeterminate and unsatisfactory samples [17, 18]. However, as we could confirm the presence of white/tan tissue macroscopically in most samples obtained using the Franseen needle, this method may overcome this limitation and reduce the necessity of ROSE. Further studies are awaited. Finally, the methods of collecting specimens after insertion of the needle were different between two needles (dry slow-pull versus 20 mL negative pressure), and the quality of the samples may have differed due to differences in techniques. The need for suction during EUS-FNA was evaluated in previous reports, but it is still controversial [19–21]. The European Society of Gastrointestinal Endoscopy technical guideline advocates the use of suction for EUS-FNA of solid mass and lymph nodes with 25-gauge or 22-gauge FNA needles [22]. However, several articles have recently reported the efficacy of slow-pull technique showing less contamination with blood which can potentially increase the diagnostic yield, especially for the core biopsy needle [2, 23, 24]. In our facility, the standard suction technique was used for the procedure using conventional FNA needle according to the guideline [22], but the slow-pull technique was used for the Franseen needle based on the previous reports and concern for increasing blood contamination given its unique shape of the tip. Further prospective study using the same method in collecting specimens will be required to validate our results.

## 5. Conclusions

In conclusion, although both needles showed high diagnostic accuracy for malignancy with no significant difference, the amount of histological specimen obtained with the new Franseen needle was significantly higher. EUS-FNB using a Franseen needle is especially useful for cases that require a certain amount of tissue; however, given the assured acquisition of core specimens from any location in the pancreas with comparable safety, and although the risk of bleeding should be kept in mind, this method will likely become a standard procedure for obtaining pancreatic tissue in the near future, taking over from EUS-FNA.

## Figures and Tables

**Table 1:** Patient characteristics.

	Group A (N = 50)	Group B (N = 36)	*P *value
Age			
median (IQR)	70.5 (60-75)	65 (57-77)	0.546
Gender			
male	30	21	
female	20	15	0.526
Size of the lesion (mm)			
median (IQR)	30 (20-39.5)	28 (23-34)	0.952
Final diagnosis			
Pancreatic adenocarcinoma	39	33	
Pancreatic metastasis	3	1	
Mass forming pancreatitis	3	0	
Neuroendocrine neoplasm	1	1	
IPMN	1	0	
ITPN	1	0	
ALL	1	0	
SCA	1	0	
SPN	0	1	0.303

IPMN: Intraductal papillary mucinous neoplasm. ITPN: intraductal tubulopapillary neoplasm.

ALL: acute lymphoblastic leukemia. SCA: serous cystic adenoma. SPN: solid pseudopapillary neoplasm.

**Table 2:** Comparison of the specimens obtained by two needles.

	Group A (N = 51 specimens from 50 patients)	Group B (N = 36 specimens from 36 patients)	*P* value
Targeted area in the pancreas			
head	21	19	
body	18	10	
tail	6	4	
uncinate process	6	3	0.744
Number of passes			
median (IQR)	2 (2-2)	2 (2-2)	0.247
Adequate histological specimen	50/51 (98%)	36/36 (100%)	0.667
Tissue sample area, mm^2^			
median (IQR)	4.07 (1.83-6.34)	1.31(0.38-3.12)	<0.0001
Sensitivity	95.3%	88.2%	0.233
Specificity	100%	100%	1
Accuracy for cancer	96.1%	88.9%	0.190

**Table 3:** Needles used according to the study period.

	Period		*P* value
	First half	Second half	
	(N = 45 sessions in 44 patients)	(N = 42 sessions in 39 patients)	
	(Oct 2016-June 2017)	(July 2017-March 2018)	
Franseen needle	19	32	
Conventional FNA needle	26	10	0.001

FNA: fine needle aspiration.

**Table 4:** Adverse events.

Event	Group A	Group B	*P* value
	(N = 51 sessions in 50 patients)	(N = 36 sessions in 36 patients)	
Overall	1 (2.0%)	0 (0%)	0.586
Bleeding	0	0	
Pancreatitis (mild)	1	0	

## Data Availability

The data used to support the findings of this study are available from the corresponding author upon request.

## Supplementary Materials

Supplementary Materials Video 1. A patient who developed bleeding with endoscopic ultrasound-guided fine needle biopsy (EUS-FNB) using a Franseen needle: the lesion was carefully observed in color Doppler mode before puncture to confirm that no major vessels were in the needle pathway. After the needle was inserted into the lesion, the specimen was collected with a dry slow-pull technique. Active bleeding was noticed under color Doppler mode along the needle tract when the needle was withdrawn. An increase in echo-free space between the pancreas and stomach was quickly identified.

## References

[1] Matsui M., Goto H., Niwa Y., Arisawa T., Hirooka Y., Hayakawa T. (1998). Preliminary results of fine needle aspiration biopsy histology in upper gastrointestinal submucosal tumors. *Endoscopy*.

[2] Ishikawa T., Mohamed R., Heitman S. J. (2017). Diagnostic yield of small histological cores obtained with a new EUS-guided fine needle biopsy system. *Surgical Endoscopy*.

[3] Adler D., Muthusamy V. R., Ehrlich D. (2018). A multicenter evaluation of a new EUS core biopsy needle: Experience in 200 patients. *Endoscopic Ultrasound*.

[4] Mukai S., Itoi T., Yamaguchi H. (2018). A retrospective histological comparison of EUS-guided fine-needle biopsy using a novel franseen needle and a conventional end-cut type needle. *Endoscopic Ultrasound*.

[5] Bang J. Y., Hebert-Magee S., Hasan M. K., Navaneethan U., Hawes R., Varadarajulu S. (2017). Endoscopic ultrasonography-guided biopsy using a Franseen needle design: Initial assessment. *Digestive Endoscopy*.

[6] Matsuzaki I., Miyahara R., Hirooka Y. (2015). Forward-viewing versus oblique-viewing echoendoscopes in the diagnosis of upper GI subepithelial lesions with EUS-guided FNA: A prospective, randomized, crossover study. *Gastrointestinal Endoscopy*.

[7] Cotton P. B., Eisen G. M., Aabakken L. (2010). A lexicon for endoscopic adverse events: report of an ASGE workshop. *Gastrointestinal Endoscopy*.

[8] Vilmann P., Jacobsen G. K., Henriksen F. W., Hancke S. (1992). Endoscopic ultrasonography with guided fine needle aspiration biopsy in pancreatic disease. *Gastrointestinal Endoscopy*.

[9] Morishima T., Kawashima H., Ohno E. (2016). Prospective multicenter study on the usefulness of EUS-guided FNA biopsy for the diagnosis of autoimmune pancreatitis. *Gastrointestinal Endoscopy*.

[10] Ishikawa T., Hirooka Y., Teman C. J. (2017). An unusual case of pancreatic metastasis from squamous cell carcinoma of the lung diagnosed by EUS-guided fine needle biopsy. *Case Reports in Gastrointestinal Medicine*.

[11] Wiersema M. J., Levy M. J., Harewood G. C., Vazquez-Sequeiros E., Jondal M. L., Wiersema L. M. (2002). Initial experience with EUS-guided trucut needle biopsies of perigastric organs.. *Gastrointestinal Endoscopy*.

[12] Larghi A., Verna E. C., Stavropoulos S. N., Rotterdam H., Lightdale C. J., Stevens P. D. (2004). EUS-guided trucut needle biopsies in patients with solid pancreatic masses: a prospective study. *Gastrointestinal Endoscopy*.

[13] Sakamoto H., Kitano M., Komaki T. (2009). Prospective comparative study of the EUS guided 25-gauge FNA needle with the 19-gauge Trucut needle and 22-gauge FNA needle in patients with solid pancreatic masses. *Journal of Gastroenterology and Hepatology*.

[14] Shah S. M., Ribeiro A., Levi J. (2008). EUS-guided fine needle aspiration with and without trucut biopsy of pancreatic masses. *Journal of Oncology Practice*.

[15] Wahnschaffe U., Ullrich R., Mayerle J., Lerch M. M., Zeitz M., Faiss S. (2009). EUS-guided Trucut needle biopsies as first-line diagnostic method for patients with intestinal or extraintestinal mass lesions. *Surgical Endoscopy*.

[16] El Hajj I. I., Wu H., Reuss S. (2018). Prospective assessment of the performance of a new fine needle biopsy device for EUS-guided sampling of solid lesions. *Clinical Endoscopy*.

[17] Larghi A., Fuccio L. (2014). Endoscopic ultrasound-guided fine needle aspiration: How to obtain a core biopsy?. *Endoscopic Ultrasound*.

[18] Iwashita T., Yasuda I., Mukai T. (2015). Macroscopic on-site quality evaluation of biopsy specimens to improve the diagnostic accuracy during EUS-guided FNA using a 19-gauge needle for solid lesions: A single-center prospective pilot study (MOSE study). *Gastrointestinal Endoscopy*.

[19] Haba S., Yamao K., Bhatia V. (2013). Diagnostic ability and factors affecting accuracy of endoscopic ultrasound-guided fine needle aspiration for pancreatic solid lesions: Japanese large single center experience. *Journal of Gastroenterology*.

[20] Saxena P., El Zein M., Stevens T. (2018). Stylet slow-pull versus standard suction for endoscopic ultrasound-guided fine-needle aspiration of solid pancreatic lesions: a multicenter randomized trial. *Endoscopy*.

[21] Wang K., Ben Q., Jin Z. (2011). Assessment of morbidity and mortality associated with EUS-guided FNA: a systematic review. *Gastrointestinal Endoscopy*.

[22] Polkowski M., Jenssen C., Kaye P. (2017). Technical aspects of endoscopic ultrasound (EUS)-guided sampling in gastroenterology: European Society of Gastrointestinal Endoscopy (ESGE) Technical Guideline - March 2017. *Endoscopy*.

[23] Lee K. Y., Cho H. D., Hwangbo Y. (2018). Efficacy of 3 fine-needle biopsy techniques for suspected pancreatic malignancies in the absence of an on-site cytopathologist. *Gastrointestinal Endoscopy*.

[24] Nakai Y., Isayama H., Chang K. J. (2014). Slow pull versus suction in endoscopic ultrasound-guided fine-needle aspiration of pancreatic solid masses. *Digestive Diseases and Sciences*.

[25] Ishikawa T., Hirooka Y., Kawashima H. (2018). Usefulness of histological assessment with Eus-guided fine needle biopsy using a new core needle in pancreatic diseases. *Gastrointestinal Endoscopy*.

